# 4228 A latent class analysis of seriously ill adults with multiple chronic conditions receiving palliative care

**DOI:** 10.1017/cts.2020.322

**Published:** 2020-07-29

**Authors:** Komal Murali, Gary Yu, John D. Merriman, Abraham A. Brody

**Affiliations:** 1 New York University; 2 New York University Rory Meyers College of Nursing

## Abstract

OBJECTIVES/GOALS: The purpose of this secondary data analysis was to identify latent subgroups of seriously ill adults based on multiple chronic conditions and mortality risk using the CCI. This study was conducted by performing a secondary analysis of data from a randomized controlled trial of seriously ill patients receiving palliative care. METHODS/STUDY POPULATION: A cross-sectional analysis of baseline CCI data was conducted. 381 seriously ill adults receiving palliative care were in the original study. Latent subgroups were identified based on the CCI by conducting a latent class analysis in MPlus. The LCA was modeled on each of the 19 disease items as binary latent predictor variables, an additional binary variable representing presence of any disease not accounted for by the CCI, and a final categorical variable representing the total CCI score divided based on clinically significant cutoffs including zero, low (> = 1-<2), moderate (> = 2-<5), and high CCI (> = 5). RESULTS/ANTICIPATED RESULTS: Three distinct latent subgroups were identified based on the CCI. Latent subgroup 1 included those with a low-moderate CCI consisting of MCC and non-Metastatic Cancers (n = 178), with 45% of this group having chronic obstructive pulmonary disease. The second two subgroups included individuals with a high CCI or a score greater than or equal to 5. Latent subgroup 2 (n = 64) was comprised of individuals with MCC and non-metastatic cancer. Latent subgroup 3 (n = 139) included individuals with metastatic cancer. DISCUSSION/SIGNIFICANCE OF IMPACT: In a sample of seriously ill adults with MCC, latent subgroups were identified consisting of individuals with low, moderate, or high CCI. The low to moderate CCI group consists of individuals with chronic conditions including COPD, congestive heart failure, myocardial infarction, cardiovascular disease. There were two subgroups with high CCI scores and the differentiating factor between the two subgroups was the presence of metastatic cancer in latent subgroup 3. The identification of latent subgroups sets the groundwork for further analyses to compare differences in symptom burden, quality of life, and functional status between groups. The findings have the potential to inform future studies seeking to better characterize seriously adults with MCC based on their disease burden and mortality risk.

